# On Rennet Wine

**Published:** 1863-09

**Authors:** George Ellis

**Affiliations:** Dublin


### ON RENNET WINE.

By Dr. GEORGE ELLIS, Dublin.

[The substance ordinarily sold under the name of Pepsine is perfectly inert. It will not coagulate milk, and has no digestive action whatever on animal substances.]

About two years since, failing to obtain any benefit from this new remedy, I had recourse to the direct preparation of a solution of gastric juice from the calf's stomach; and so gratifying has been the result, so satisfactory and remarkable its effects as a remedy in gastric derangements, that I wish to communicate to the profession the mode of preparation which I have found the most convenient and the best for every purpose.

Take the stomach of a calf fresh from the butcher; cut off about three or four inches of the upper or cardiac extremity, which, containing few glandular follicles, may be thrown away. Slit up the stomach longitudinally; wipe it gently with a dry napkin, taking care to remove as little of the clean mucus as possible. Then cut it into small pieces (the smaller the better,) and put all into a common wine bottle. Fill up the bottle with good sherry, and let it remain corked for three weeks; at the end of this time it is fit for use.

*Dose.*—One teaspoonful in a wineglassful of water immediately after meals.

*Test of Quality.*—One teaspoonful will solidify, to the consistency of blanc-mange, in from one to two minutes, a cup of milk (say eight to ten ounces,) at the temperature of 100° Fahr. In this action on the caseine of the milk, it may be said that the wine alone would have some effect, but wine will not solidify milk, nor will it curdle it at all, except at a much higher temperature and in much larger proportion than the above.

This preparation, which I propose to call "Rennet Wine," has many advantages over the watery infusion of rennet which is obtained from the salted and dried calf's stomach, (used largely in cheese making.) The latter is also a good preparation, solidifying milk in the same way while it remains fresh; but it is much more troublesome in the making, and in warm weather it soon begins to react on the animal matters contained in it, and becomes spoiled. For these reasons, it cannot conveniently be used in medical practice. Rennet wine, on the contrary, is so easily made, requiring no salting or drying of the stomach, is so inexpensive, and can so readily be prescribed in private and in hospital practice, that I have little doubt, when known, it will become one of the most valued remedial articles in the hands of the profession.

I recommend the employment of good sherry, because this wine has sufficient body to keep the infusion perfectly sound for any length of time, and is not so strong in alcohol as to suffer any apparent loss of solvent power in taking up the active principle of the rennet.

To the physiologist, it is unnecessary to say, that this remedy should be given after or during, not before, meals. A single dose, given daily after dinner, I have found quite sufficient in the general run of cases requiring it. How this small quantity can act so speedily and effectively it is, perhaps, not easy to explain, when we consider the large supply of the gastric secretion necessary for the thorough digestion of an ordinary meal. The action is, probably, due to those indirect chemical changes, called catalytic transformations, which some organic substances, by their mere presence and contact, induce in each other and in other proximate principles; and thus, perhaps, the conversion of a small portion of food into healthy albuminose by this small quantity of sound gastric juice may induce the same healthy action throughout the stomach content during the entire process of stomach digestion. It is at least equally difficult to explain the action and rapid extension of ferments, generally, in their appropriate solutions.

I have often been forcibly struck by the magical effect of this small dose in removing offensive odor from the breath of young persons,—a distressing symptom, sometimes aggravated rather than relieved by purgative medicine; and I may also mention, that in one of these cases cod-liver oil was easily tolerated afterwards, though never before. It would be a mistake, however, to suppose that the oil is at all acted on by the gastric fluid. The oil globules of coagulated milk are seen, under the microscope, unchanged, though imbedded in the solidified caseine; and the digestion of oil, taking place only after passing the orifices of the pancreatic and biliary ducts, is entirely intestinal; but intestinal digestion itself must surely be influenced essentially by the healthy preparatory action of the stomach secretion on the albuminous compounds presented to it, and thus the digestion of oils and fatty matters, though not even commenced in the stomach, may be facilitated by their being mingled with the products of healthy gastric action, when submitted to the succeeding operations of the pancreas and liver.—*Medical Times and Gazette.*

